# Goitered Gazelle *Gazella subgutturosa* Responded to Human Disturbance by Increasing Vigilance Rather than Changing the Group Size

**DOI:** 10.3390/biology11081236

**Published:** 2022-08-19

**Authors:** Wenxuan Xu, Muyang Wang, David Blank, António Alves da Silva, Weikang Yang, Kathreen E. Ruckstuhl, Joana Alves

**Affiliations:** 1 State Key Laboratory of Desert and Oasis Ecology, Xinjiang Institute of Ecology and Geography, Chinese Academy of Sciences, Urumqi 830011, China; 2 The Specimen Museum of Xinjiang Institute of Ecology and Geography, Chinese Academy of Sciences, Urumqi 830011, China; 3 Mori Wildlife Monitoring and Experimentation Station, Xinjiang Institute of Ecology and Geography, Chinese Academy of Sciences, Mori 831900, China; 4 Sino-Tajikistan Joint Laboratory for Conservation and Utilization of Biological Resources, Urumqi 830011, China; 5 Research Center for Ecology and Environment of Central Asia, Bishkek 720001, Kyrgyzstan; 6 Centre for Functional Ecology (CFE), TERRA Associate Laboratory, Department of Life Sciences, University of Coimbra, 3000-456 Coimbra, Portugal; 7 Department of Biological Sciences, University of Calgary, 2500 University Drive Northwest, Calgary, AB T2N 1N4, Canada

**Keywords:** Kalamaili, mining activities, *gazella subgutturosa*, human disturbance

## Abstract

**Simple Summary:**

Wild animals perceive nonlethal human activities as a potential threat, and they respond to human disturbances in a variety of ways: changes in group size and time-investment on vigilance are common behaviors. In China, nature reserves are generally divided into three functional zones: the core zone, the buffer zone, and the experimental zone, which represent low, medium, and high human disturbance levels, respectively. However, mining developments in the three zones pose threats to goitered gazelle living in the Kalamaili Nature Reserve, China. In this study, we found that human disturbances have larger effects on smaller-sized, single-sex groups rather than larger-sized, mixed-sex groups. Goitered gazelle spend more time being vigilant in the experimental zone than in the other two zones. In the experimental zone, a greater linear decrease with respect to group size was observed. Our results indicate that intensive human disturbances in the core and buffer zones brought goitered gazelle and humans closer together, which induced changes in vigilance and grouping patterns of this social species. Ultimately, this may have negative effects on the fitness and survival of the species.

**Abstract:**

Nonlethal human disturbances have been confirmed to have a negative effect on wildlife in a variety of ways, including on behaviors. In many studies, the impact of human disturbances on gregarious species of wildlife is assessed through changes in their social organization and vigilance. In our study in the Kalamaili Nature Reserve, China, we wanted to estimate the impacts of differing levels of human disturbances on two different group types (mixed-sex and all-male) of goitered gazelle, *Gazella subgutturosa*, living in three functional zones (experimental, buffer, and core zones), which represent high, medium, and low human disturbance levels, respectively. In addition, we studied the time spent vigilant as a function of group size with different levels of human disturbances in the three zones. In general, mixed-sex gazelle groups were of similar sizes in the three different zones, while all-male groups slightly differed in their sizes between the experimental and buffer zones. This may indicate that human disturbances have varying effects on the different group types, with smaller-sized, single-sex groups being more significantly affected by human disturbances than larger-sized, mixed-sex groups. Goitered gazelle showed higher vigilance levels in the experimental zone than in the two other zones. A trend of decreasing vigilance varying linearly with group size was also found in the three zones, and the rate of decrease was higher in the experimental zone. Increased habitat fragmentation and human activities brought goitered gazelle and humans closer together in the core zone. Therefore, stopping mining activities and returning the reserve to a continuous habitat with fewer environmental disturbances is the best way to establish and protect a stable population of this endangered species of gazelle.

## 1. Introduction

Increased fragmentation and accessibility of natural areas brings wildlife into closer contact with human activities. In some cases, this contact is merely a nuisance-type disturbance and does not pose a direct threat to the animals. Frequently, however, wildlife also perceives nonlethal human activities as a potential threat, which is also known as the “human-caused predation risk” hypothesis [1,2,3]. Human disturbances may lead to behavioral changes in individuals and may ultimately affect the demography and social organization of a species [4,5]. Therefore, in conservation efforts, it is important to understand the impact of human disturbance on animal behavior and/or on the group composition of social animals [6,7].

Both human disturbance and predation risk elicit similar responses in wildlife, such as increasing group sizes and vigilance rates [8,9,10]. In many studies, the impact of human disturbances on wildlife is assessed by comparing the group size, composition, and vigilance behavior of gregarious species between high and low disturbance areas. For example, an increase in the amount of time spent foraging in response to human disturbances has been reported for wildebeest (*Connochaetes taurinus*), zebra (*Equus quagga burchelli*) [11], khulan (*Equus hemionus*) [12], Père David’s deer (*Elaphurus davidianus*) [13,14], and bighorn sheep (*Ovis canadensis*) [15]. However, another study on 19 mammals on this subject showed no substantial change in vigilance in the presence of disturbances [16]. Animals typically form larger groups in high compared to low disturbance areas [17,18]. The formation of larger groups benefits animals by reducing potential predation risk via shared vigilance (known as the many eyes hypothesis) [19]; early detection; predator confusion [20]; and by decreasing a single individual’s danger of being targeted (the risk-dilution hypothesis) [21], which is also known as the ‘group size’ effect [19]. However, human disturbances and group size could have an additive or interactive effect on individual or group vigilance behavior [22,23]. That is, higher levels of human disturbances would dampen the benefit from increases in group size, compared to those in the undisturbed core areas [12].

The goitered gazelle is a mid-sized antelope that lives in the semi-deserts and deserts of the Asian continent. This species lives in sexually-segregated groups [24,25] of typically 2–5 individuals across its distribution range [26,27,28]. Goitered gazelle in the Kalamaili Mountain Ungulate Nature Reserve (hereafter KNR) suffer from habitat degradation and fragmentation caused by human disturbances, such as roads, mining, and competition with grazing livestock [29]. These forms of human activity are known to have a negative effect on the social structure and vigilance behaviors of wild ungulates [12,15,30,31].

In China, nature reserves can be divided into three functional zones: the core zone, the buffer zone, and the experimental zone. The core zone is the concentrated distribution area of rare and endangered animals and plants in the reserve, which is forbidden to enter and is absolutely protected. A buffer zone is a certain area outside of the core zone that is used to alleviate external pressure and prevent the impact of human activities on the core zone. Scientific research activities can be carried out in this area. The experimental zone is located around the buffer zone, which is a multi-purpose area. In our study, we address whether and how goitered gazelle adjust their behaviors, their group size, and composition in the three functional zones of the KNR. We predict a larger group size in the experimental zone and buffer zone, which are intensively disturbed areas, compared with the core zone (least disturbed). Furthermore, we predict gazelle will spend a higher proportion of their time in vigilance in more human-disturbed zones despite a predicted decrease in vigilance with increasing group size. Regarding sex, and since our study was mostly conducted during summer, we expect higher vigilance in females due to their reproductive status than in males.

## 2. Materials and Methods

### 2.1. Study Area

**Figure 1:**
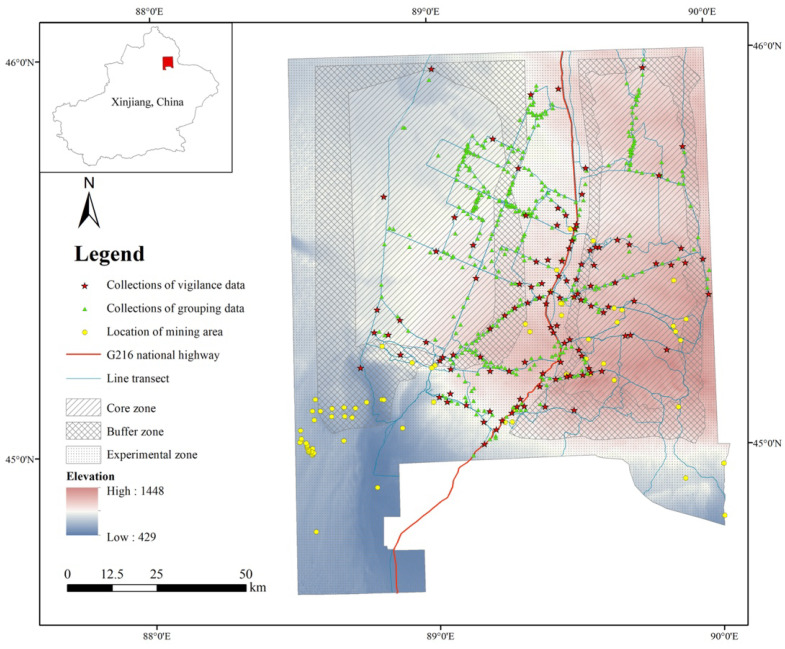
Location and functional zones of the Kalamaili Mountain Ungulates Nature Reserve. Transect lines and the distribution of the mining area is also shown in the figure. The core zone is highlighted with a striped pattern, the buffer zone with a hatched pattern, and the experimental zone with an area showing horizontal lines. Blue areas represent low elevations and red areas represent higher elevations. Please see the legend for more details.

This study was conducted in the KNR (44°36′–46°00′ N, 88°30′–90°03′ E), located in the eastern part of the Junggar Basin, Xinjiang, China (Figure 1). The elevation of the KNR is between 600 and 1700 m above sea level. This region has a harsh continental climate with an average yearly temperature of +1.99 °C. Winters are cold and long; summers are hot and short. The average annual rainfall is around 186.8 mm. Vegetation cover is quite sparse and consists mostly of desert and dwarf shrubs from the families Chenopodiaceae, Ephedraceae, Tamaricaceae, and Zygophyllaceae. The most common desert tree is the saxaul (*Haloxylon ammodendron*). Common shrubs are *Anabasis salsa*, *Atraphaxis frutescens*, *Calligonum mongolicum*, *Ceratocarpus arenarius,* and *Ceratoides latens*. Around 6628–19,677 goitered gazelle live in the KNR [32]. The natural predation risk is considered very low, since wolves, the main predators of goitered gazelle, exist in very low numbers. 

Before 2005, the main human activity was from herdsmen, who stayed in the KNR only for the winter months; from November to March each year. Our study was conducted during the summer, so the effect of herdsmen was not included. Since 2007, however, human activities have increased, including the development of coal and rock mining and oil operations, which are mostly in the experimental and buffer zone, though some are also located in the core zone of the KNR. From 2015 onwards, all human activity was halted in the reserve, except for the presence of herdsmen. Thus, the animals in all the three functional zones of the KNR were faced with very high levels of human disturbances from 2007 to 2014. The increase in human disturbances in three zones, especially in the core zone, would change the behavior of wild animals, including goitered gazelle. During this period, the population size of khulan, another ungulate common to the KNR, decreased to half of its pre-2007 level [33]. Although the goitered gazelle population in the KNR was not counted at the time, it seems likely that human activity was a major threat to all wildlife, including the goitered gazelle.

### 2.2. Data Collection

From 2008 to 2012, data for evaluating group composition were collected during the summers (June–August). In total, we spent 47 days in the field, i.e., 10, 9, 10, 7, 11 days in each summer. The survey was conducted using transects by car at low speed (less than 30 km/h) and covered the whole reserve. The length of daily transects varied between 30 and 53 km. We searched for goitered gazelle using binoculars (magnification 8×) and telescopes (magnification 20×–60×) every 3–5 km. We recorded the group size, composition of each observed group, and the sex of each individual. As it was not possible to determine exact ages for both males and females, we distinguished individuals as adults, sub-adults, or fawns. Identifications were made based on the body size, length of horns, muzzles, and neck coloration (see details in [24]). Individuals were defined as members of a group when the distance between them were less than 50 m [34]. Individuals farther than 50 m were not considered part of the group. We distinguished between female groups, male groups, and mixed-sex groups. Female groups included one or more adult females plus young and/or yearlings, but no adult males. Male groups included one or more adult males plus sub-adult males. Mixed groups included at least one adult of each sex. In total, 497 groups were observed, including 146 groups in the buffer zone, with a range of group sizes from 1 to 31 individuals; 81 groups in the core zone, with a range of group sizes from 1 to 24; and 270 groups in the experimental zone, with a range of group sizes from 1 to 54.

The vigilance behavior of goitered gazelle was investigated using the focal animal sampling method during daylight hours through the summers of 2008–2011. Vigilance was defined when a gazelle was scanning its surroundings. Target groups were randomly selected and observed using a telescope (magnification 20 × 60). We collected data from as many groups as possible, with only a few individuals from the same group being observed to reduce the possibility of pseudo-replication. Since the vigilance competence of fawns is not usually well-developed, we only randomly selected focal animals of adults and made direct observations. Each gazelle was observed over a 10 min period. Samples that lasted less than 10 min because of a subject leaving the group or the group size changing were excluded from our analyses. Using Aigo voice recorders, we recorded all behaviors of the focal animal, and the data were later transcribed to numerical files by replaying our audio recordings. During the whole study, we recorded 541 focal observations from 262 groups, including 181 samples from 65 groups (group size ranges from 2 to 27) in the core zone, 123 samples from 49 groups (group size ranges from 2 to 21) in the buffer zone, and 238 samples from 148 groups (group size ranges from 2 to 21).

### 2.3. Data Analysis

To test our predictions, a general linear mixed model (GLMM) with a negative binomial distribution was used to analyze the effects of functional zone, group type, and its interaction on group sizes. The date of observation was included as a random factor to further control for any potential pseudo-replication resulting from multiple observations of the same individuals over time.

Regarding vigilance, a GLMM with a negative binomial distribution was used to evaluate the potential effect of functional zone, group size, and sex (and their interactions) on the number of seconds spent vigilant. Group ID was included as a random factor.

The independence of predictor variables was confirmed through correlation analysis and variance inflation factors [35]. For each dependent variable, pairwise multiple comparisons were performed using sequential Bonferroni correction. Model validation was performed for each GLMM on the residuals by checking for heteroscedasticity, normality, and influential observations. The results are expressed as estimated means and 95% confidence intervals (CI). All statistical tests were considered significant when *p* < 0.05. The statistical analyses were performed using R version 4.0 [36].

## 3. Results

### 3.1. Difference in Group Size among Functional Zones

**Figure 2:**
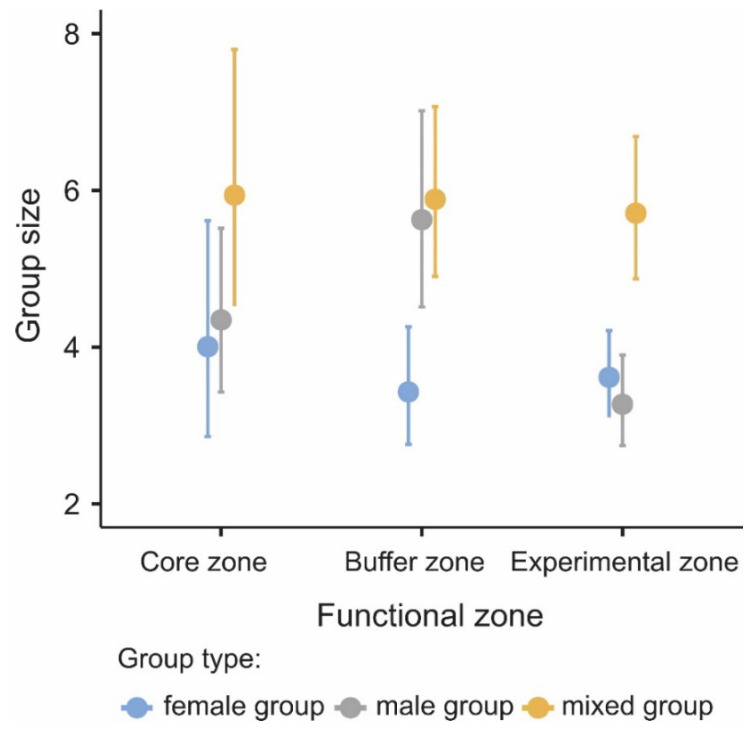
Group size of female (blue dots), male (gray dots), and mixed-sex groups (yellow dots) of goitered gazelle occurring in the core, buffer, and experimental zones. The values represent the estimated means from the general linear mixed model, and the bars represent the 95% confidence intervals.

Globally, the group size of goitered gazelle in this study was 4.86 ± 0.21. Our results show a significant interaction between the functional zone and group type (χ^2^ = 11.92, *p* = 0.018; Table 1), which is derived from the differences in the size of male groups between the buffer zone (5.63 ± 0.63) and the experimental zone (3.27 ± 0.29) (Figure 2). For the other group types, no difference was found in their size in each of the functional zones (Figure 2).

### 3.2. Difference in the Level of Vigilance among Functional Zones

**Figure 3:**
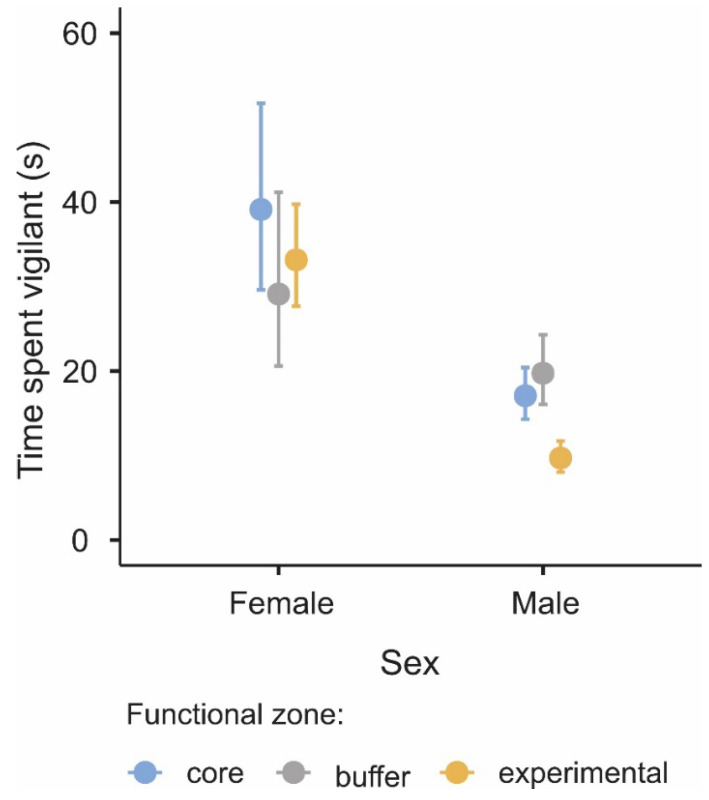
Influence of sex of goitered gazelle on the time spent vigilant (in seconds) in the core (blue dots), buffer (gray dots), and experimental zones (yellow dots). The values represent the estimated means from the general linear mixed model, and the bars represent the 95% confidence intervals.

The vigilance levels of goitered gazelle were affected by functional zone, group size, sex, and the interaction terms between functional zone and sex and functional zone and group size (Table 2). Goitered gazelle females were generally vigilant for longer than males (β_males_ = −0.815, z = −8.689, *p* < 0.001), except in the buffer zone (z = 2.038, *p* = 0.623) (Figure 3). Goitered gazelle showed different times spent on vigilance in the three zones (χ^2^ = 12.7, *p* = 0.002, Table 2). However, the effect of functional zones on vigilance was different for females and males, with a significant interaction between functional zone and sex (χ^2^ = 13.8, *p* = 0.001, Table 2). Females were similarly vigilant in all the three zones, while males showed lower vigilance in the experimental zone compared to the core (z = 4.301, *p* < 0.001) and buffer zones (z = 4.989, *p* < 0.001) (Figure 3). 

**Figure 4:**
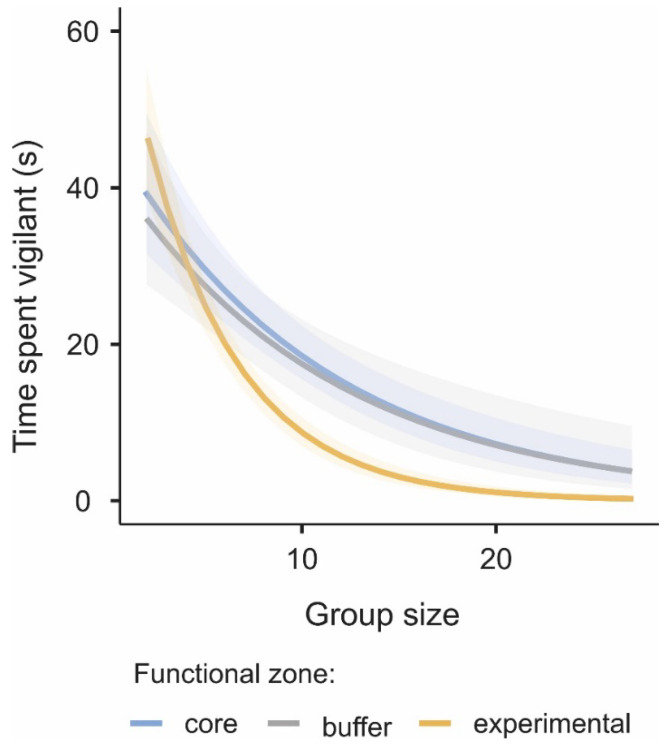
Effect of goitered gazelle group size on the time spent vigilant (in seconds) in the core (blue line), buffer (gray line), and experimental (yellow line) zones. The lines represent the estimated means from the general linear mixed model, and the shadows represent the 95% confidence intervals.

Considering the effect of group size on the time spent vigilant, there was a clear decrease in the proportion of time spent vigilant with increasing group size and more strongly so in the experimental zone (β = −0.116, z = −5.114, *p* < 0.001; Figure 4). 

## 4. Discussion

Grouping and vigilance are complementary responses of antipredator behavior in ungulates [37]. However, different ungulate species may show different responses and do not synchronously adjust these two behaviors. When faced with human disturbances, some tend to increase their vigilance, while other species prefer to aggregate into larger groups without increasing vigilance [12,38,39]. 

We predicted that goitered gazelle would increase their group size in the experimental zone and buffer zone, which represent higher intensities of human disturbances than the core zone. Our results instead showed that goitered gazelle respond to human disturbances mainly through increased vigilance rather than increasing group sizes. These results are similar to those found in the Kazakhstan population of the same species, where gazelle changed their vigilance levels in response to human disturbances before changing their group sizes [40]. However, other species, such as impala (*Aepyceros melampus*) or key deer (*Odocoileus virginianus clavium*), increased group sizes instead of changing vigilance duration when faced with human disturbance [23,38,39].

Although we found a significant interaction between the functional zone and group type of goitered gazelle, it derived only from differences in the size of male groups between the buffer zone and the experimental zone. Mixed-sex groups and female groups, however, are of the same size in the three functional zones. Human activities differentially affect the size of different types of groups, with smaller, single-sex groups generally being more affected when faced with high levels of human disturbances [41]. This effect should be especially pronounced in species that naturally form small-sized groups [42]. For example, male groups of common eland (*Tragelaphus oryx pattersonianus*) are relatively small compared to other group types but have increased in areas with intensification of human disturbances [42]. The larger size of male groups in the buffer zone probably occurs because they increase their group size as an anti-predatory strategy, while in the experimental zone, they choose the strategy of being solitary to attract less attention to themselves.

Numerous studies have confirmed that the size of mixed-sex groups of ungulates are generally larger than single-sex groups [42,43,44,45], including goitered gazelle [26,27,28]. The most important benefit of staying in larger mixed-sex groups, other than mating opportunities, could, in fact, be that individuals can afford to spend less time being vigilant than in smaller groups. However, the forage conditions for a species such as the goitered gazelle, which lives in a Gobi habitat with sparse vegetation cover and scattered patches of plants, do not allow them to form larger groups. Therefore, there is likely a trade-off between safety in numbers and increased competition for food, with more restrictive environments selecting against the formation of larger groups. In contrast, species such as the zebra, which often live in conditions with an abundance of grass, can increase their group sizes more readily [37].

Accordingly, the increased human disturbance level from 2008 to 2012 in the KNR did not lead to increasing group sizes in the goitered gazelle population compared to levels previously reported between 2005 and 2007 [26], when human disturbance was lower than in our study [12,33].

Partly in line with our predictions, only males changed their investment in vigilance when faced with diverse levels of human activity in each of the three functional zones. The ‘human-caused predation risk’ hypothesis states that animals react to a perceived threat from human activities [1] by devoting more time to vigilance in the area with greater human disturbances (or predation risk) [3,46,47]. This is true for goitered gazelle males in our study since they spent less time being vigilant in the experimental zone than in the buffer and core zones. A previous study on khulan also found that human disturbance increased vigilance levels similar to that of goitered gazelle [12]. The core zone, which had restricted access compared to the buffer and experimental zone, was generally established for the protection of wildlife. However, when we conducted our research, a lot of human activity appeared in the core zone due to energy exploitation in the area, which led to an increase in habitat fragmentation for both khulan and goitered gazelle. In such fragmented landscapes, both species have to spend more time being vigilant because of longer flight distances [12], which led to an increase in vigilance in the core zone.

Human disturbance (or predation risk) and group size are two key determinants of vigilance. Vigilance is expected to increase when the risk posed by human disturbances or predators increases, and to decrease in larger groups that provide more safety against predators. If human disturbances and group size interact, then the magnitude of the ‘group size’ effect on vigilance would vary depending on the level of disturbance experienced [23,48]. In the current research, we found an interaction between group size and disturbance level (functional zone): vigilance levels decreased as group size increased in all zones, but with a higher magnitude in the experimental zone. Reviewing the literature in terms of interaction effects between potential risk and group size on vigilance, Beauchamp [48] noted the majority of studies found no statistically significant interaction effect between vigilance and group size in mammals when facing human disturbances: for example, *Capra nubiana* [49], Equus kiang [50], *Gazella thomsonii* [51], *Aepyceros melampus*, *Tragelaphus strepsiceros*, and *Hippotragus niger* [52]. There is no doubt that human disturbances, including roads and mining, result in increased habitat fragmentation, which brings humans into closer contact with gazelle even in the core and buffer zone, adding potential threats to these animals. When human disturbances are stronger in the core and buffer zones, gazelle probably need to increase their vigilance investment. Such an increase in vigilance may occur primarily in large groups because the level of vigilance is already high in small groups. However, gazelle may have become accustomed to non-lethal human disturbance before the mining development appeared in the experimental zone of the KNR, and they thus behave similarly whether there is human disturbance from mining developments or not [23]. 

Human disturbances generally pose an indirect threat to wildlife through an increase in habitat fragmentation, which, in turn, brings more humans within closer contact with wildlife. Such disturbances may induce behavioral changes in animals, such as vigilance and grouping patterns, both of which are important for social species, and ultimately may have negative consequences on fitness and survival. However, it is difficult to directly observe any changes in fitness caused by the adjustment of goitered gazelle behavior, which would require much longer-term monitoring and research.

## 5. Conclusions

Our study provides an opportunity to examine whether wild animals alter one behavior and/or the other when disturbances become more frequent. It contributes to gaining new knowledge about the behavior adjustment and adaptation under a background of human dominant environments. Globally, goitered gazelle adjust their behaviors among the three zones mostly in vigilance level rather than group size. Such changes in vigilance patterns caused by human disturbances in goitered gazelle possibly affect the survival of the species. Halting the mining activities in the reserve and reducing environmental disturbances is the best way to establish and protect a stable population of this endangered species.

## Figures and Tables

**Table 1:** Influence of functional zone and group type on group size of goitered gazelle using a GLMM.

Parameters	χ^2^	df	*p*
Functional zone	5.85	2.00	0.054
Group type	29.96	2.00	<0.001
Functional zone × Group type	11.92	4.00	<0.018

Marginal R^2^: 0.423; Conditional R^2^: 0.603.

**Table 2:** Influence of functional zone, sex, and group size in the time spent vigilant in goitered gazelle, using a GLMM.

Parameters	χ^2^	df	*p*
Functional zone	12.7	2.00	0.002
Group size	157.2	1.00	<0.001
Sex	75.5	1.00	<0.001
Functional zone × sex	13.8	2.00	0.001
Group size × functional zone	29.5	2.00	<0.001

Marginal R^2^: 0.127; Conditional R^2^: 0.271.

## Data Availability

Data sharing is not applicable to this article.
